# Pentacyclic Triterpenoids with Nitrogen-Containing Heterocyclic Moiety, Privileged Hybrids in Anticancer Drug Discovery

**DOI:** 10.3390/molecules26092401

**Published:** 2021-04-21

**Authors:** Vuyolwethu Khwaza, Sithenkosi Mlala, Opeoluwa O. Oyedeji, Blessing A. Aderibigbe

**Affiliations:** Department of Chemistry, University of Fort Hare, Alice Campus, Alice 5700, Eastern Cape, South Africa; vkhwaza@ufh.ac.za (V.K.); smlala@ufh.ac.za (S.M.); ooyedeji@ufh.ac.za (O.O.O.)

**Keywords:** pentacyclic triterpenoids, *N*-heterocycles, hybrids, derivatives, anticancer, oleanolic acid

## Abstract

Pentacyclic triterpenoids are well-known phytochemicals with various biological activities commonly found in plants as secondary metabolites. The wide range of biological activities exhibited by triterpenoids has made them the most valuable sources of pharmacological agents. A number of novel triterpenoid derivatives with many skeletal modifications have been developed. The most important modifications are the formation of analogues or derivatives with nitrogen-containing heterocyclic scaffolds. The derivatives with nitrogen-containing heterocyclic compounds are among the most promising candidate for the development of novel therapeutic drugs. About 75% of FDA-approved drugs are nitrogen-containing heterocyclic moieties. The unique properties of heterocyclic compounds have encouraged many researchers to develop new triterpenoid analogous with pharmacological activities. In this review, we discuss recent advances of nitrogen-containing heterocyclic triterpenoids as potential therapeutic agents. This comprehensive review will assist medicinal chemists to understand new strategies that can result in the development of compounds with potential therapeutic efficacy.

## 1. Introduction

Plants are considered common alternative for the treatment of cancer in most countries, and over 3000 plant species worldwide have anticancer properties [1,2] Plant-derived natural compounds have attracted the attention of many researchers due to their wide range of biological activities with various target sites and less toxic effects to normal cells [3]. Pentacyclic triterpenoids (PTs) are the most significant and well-studied phytochemicals. PTs have been screened for biological activities, and most of them displayed promising in vitro and in vivo potency [4]. A number of PTs and their derivatives are known to exhibit a wide range of biological activities such as anticancer [5,6,7], anti-HIV [8,9,10], anti-inflammatory [11,12], antimalarial [13], hepatoprotective [14,15], antimicrobial [16,17] antioxidant [18,19] and antidiabetic activities [20]. Currently, there is an increase in the number of patents being issued to protect new PTs with potential therapeutic effects, especially anticancer and antiviral [21,22,23,24]. Although PTs have such interesting biological properties, several disadvantages (e.g., low water solubility, selectivity, poor bioavailability, and short half-life) that hinder their potential therapeutic application in clinical use have been reported. Currently, the pharmacokinetic profile of PTs has not been thoroughly characterized, although numerous in vivo studies have been reported [25]. A vast number of studies have been performed to improve their pharmacological activities by introducing heterocyclic scaffolds into the PT’s structure [26]. On the other hand, PTs can be a potential lead for the development of novel drugs. As a result, their structural modification has been reported as a promising strategy to enhance their pharmacological activity [27]. Among these PTs, derivatives with nitrogen-containing heterocyclic scaffolds play a major important role. Nitrogen-containing heterocyclic compounds are the most frequently used therapeutic agents. Firstly, these derivatives are usually more stable with good bioavailability. Additionally, heterocyclic derivatives have significant absorption bands upon UV light irradiation, which simplifies their detection in many in vivo studies and subsequent clinical testing; it helps to define the impurity profile in drug manufacture. Heterocyclic derivatives are easily prepared in selectively labeled form due to the good availability of building blocks in an isotopic form (^15^N, ^33^S, etc.). The above-mentioned advantages of heterocyclic derivatives give them great potential for use in pharmaceutical practice. These compounds have been used to develop various organic synthetic procedures and have a wide range of therapeutic applications [28,29]. Nowadays, triterpenoids with nitrogen-containing heterocyclic scaffolds have been extensively studied for their potential anticancer activities. In this review, we discuss synthesized triterpenoid hybrid compounds with fused nitrogen-containing heterocyclic ring(s) such as Triazole, Pyrazole, Indole, Piperazine, and Aminoquinolines with potential anticancer activity.

## 2. Anticancer Activity of Pentacyclic Triterpenoids

**Figure 1:**
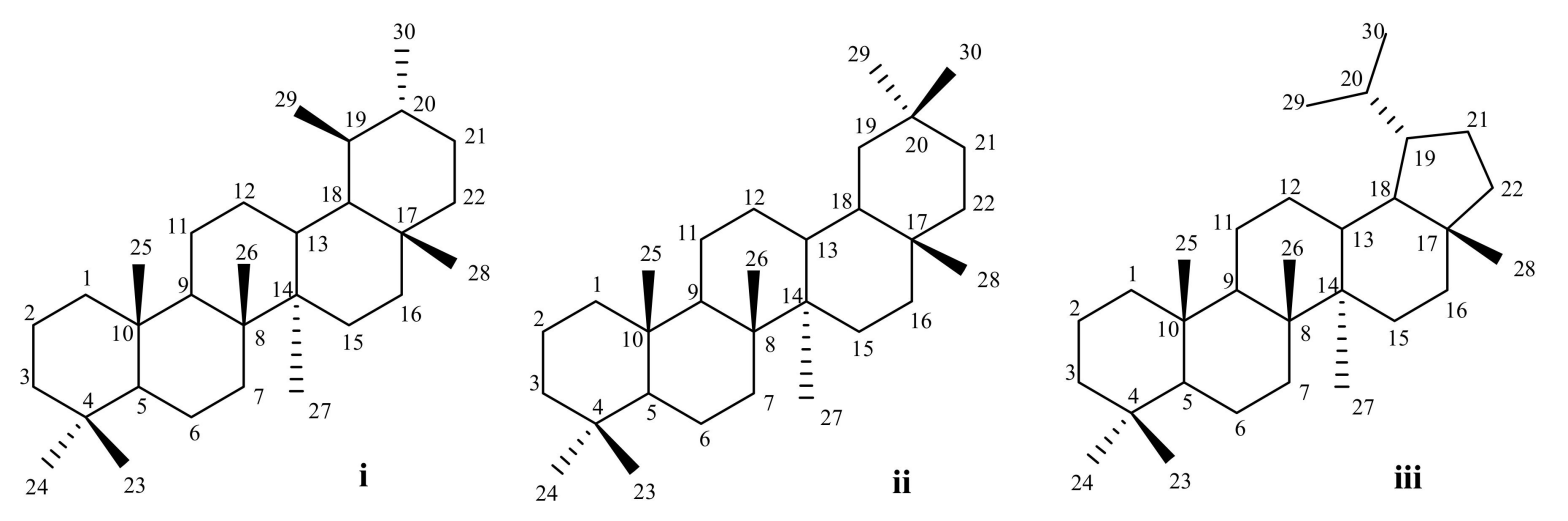
Three different types of triterpenoid skeletons described in this review with carbon numbering: ursane (**i**), oleanane (**ii**), and lupane (**iii**).

The morbidity and mortality rates caused by cancer are rapidly increasing globally. According to the 2018 statistics reported by the World Health Organisation (WHO), about 18.1 million cases of cancer were recorded, and 9.6 million cancer-related deaths. This means 1 in 6 deaths worldwide is due to cancer [30]. The use of chemotherapeutic agents in cancer treatment is always associated with side effects affecting organs and systems in the human body [31]. Many factors are responsible for the high rate of cancer, including age, population growth, lifestyle, etc. [32]. Cancer is known as the abnormal proliferation of cells, which can later invade different body parts. The currently used strategies for cancer treatment are chemotherapy, hormone therapy, and a combination of surgery and radiotherapy. However, some of the limitations associated with the conventional drugs used for the treatment of cancer are the unselective targeting of cells, multi-drug resistance, relapse of cancer, and poor outcomes [33]. The search for suitable and easily accessible treatment(s) for cancer, scientists and researchers around the world have turned their attention to natural products, phytochemicals, and their derivatives due to their therapeutic effects in the treatment of various diseases. Over 75–80% of the world’s population consume medicinal plants for their health care [34]. More than 40% of conventional drugs originate from active natural products [35]. PTs are at the forefront of phytochemicals with anticancer activities as they act as a modulator of various molecular targets in multiple signaling pathways. Triterpenoids are functionalized triterpenes, which can be synthesized from plants through cyclization of squalene intermediate with six isoprene units [36]. From a biological viewpoint, most of the important triterpenoid structures are ursane, oleanane, and lupane triterpenoids (Figure 1) [37]. Many studies on PTs revealed their cytotoxicity on a variety of tumor growth cells without showing any lethality on normal cells [20]. Some of the synthetic triterpenoid derivatives, namely 2-cyano-3,12-dioxooleana-1,9(11)-dien-28-oic acid (CDDO) and its C-28 methyl ester (CDDO-Me), are already in Phase I clinical trials in cancer patients [25,38]. Betulinic acid derivative called bevirimat exhibited a significant and clinically relevant reduction of the viral load in ART-experienced and naive patients [39]. However, other clinical trials of bevirimat also revealed a high baseline drug resistance attributed to naturally occurring polymorphisms in HIV-1 Gag [40]. In terms of anticancer activity, PTs promote the cell apoptosis, which is one of the most significant mechanisms to suppress tumor. For example, PTs can trigger apoptosis by Interfering with the mitochondrial function of tumor cells [41]. In addition, they can also induce apoptosis by up-regulating p53 and caspase-3 gene expressions and suppressing the NF-κB mediated activation of Bcl-2 in B16F-10 melanoma cells [42]. PTs can enhance tumor necrosis factor (TNF)-related apoptosis-inducing ligand (TRAIL) [43,44]. Other studies revealed that PTs inhibit the activation of STAT3 induced by interleukin-6 in DLD-1 colon cancer cells [45].

## 3. Triazole

1,2,3-triazole derivatives has attracted much attention due to their interesting pharmacological activities such as anticancer [46,47,48], antimalarial [49], antibacterial [50,51], antifungal [52,53,54], anti-inflammatory [55], and antiviral activities [56,57]. In addition, some 1,2,3-triazole-based derivatives are under clinical evaluation for their potential use in cancer treatment [58]. Hybrid molecules can overcome drug resistance and reduce side effects since hybrids containing two or more different compounds can also display multiple modes of action. Hybrid molecules containing 1,2,3-triazole moiety and other anticancer agents are potential anticancer agents that can display low toxicity and high therapeutic effects against drug-resistant cancers. In recent years, PTs such as betulinic, oleanolic, and ursolic acid were found to possess potent anticancer activity with various molecular targets. The attachment of these phytochemicals with 1,2,3-triazole moiety is a promising approach to developing novel anticancer agents that are effective against drug-resistant cancers due to their distinct mechanisms.

### 3.1. Anticancer Effects of Betulinic Acid-based 1,2,3-Triazole Molecules

**Figure 2:**
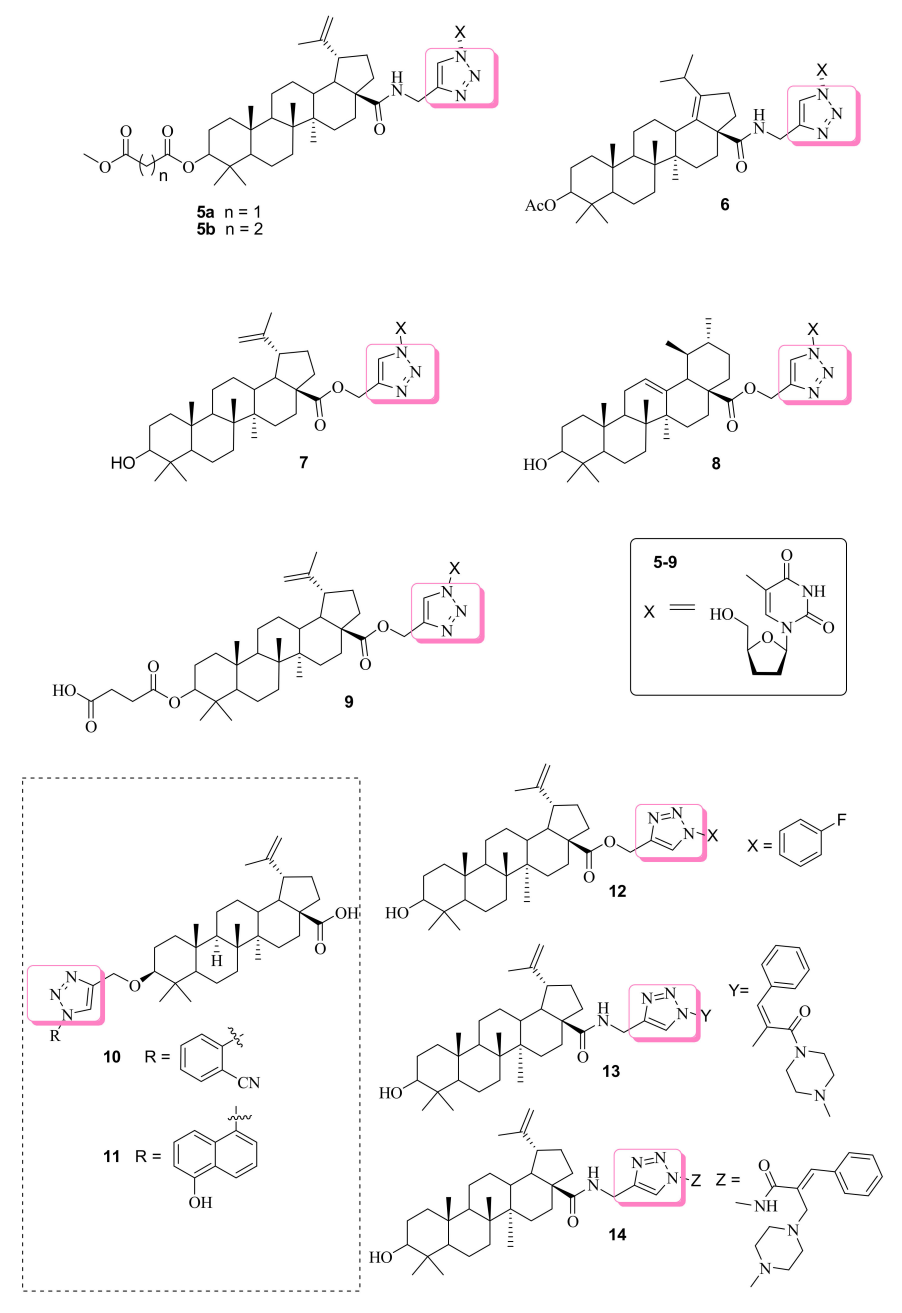

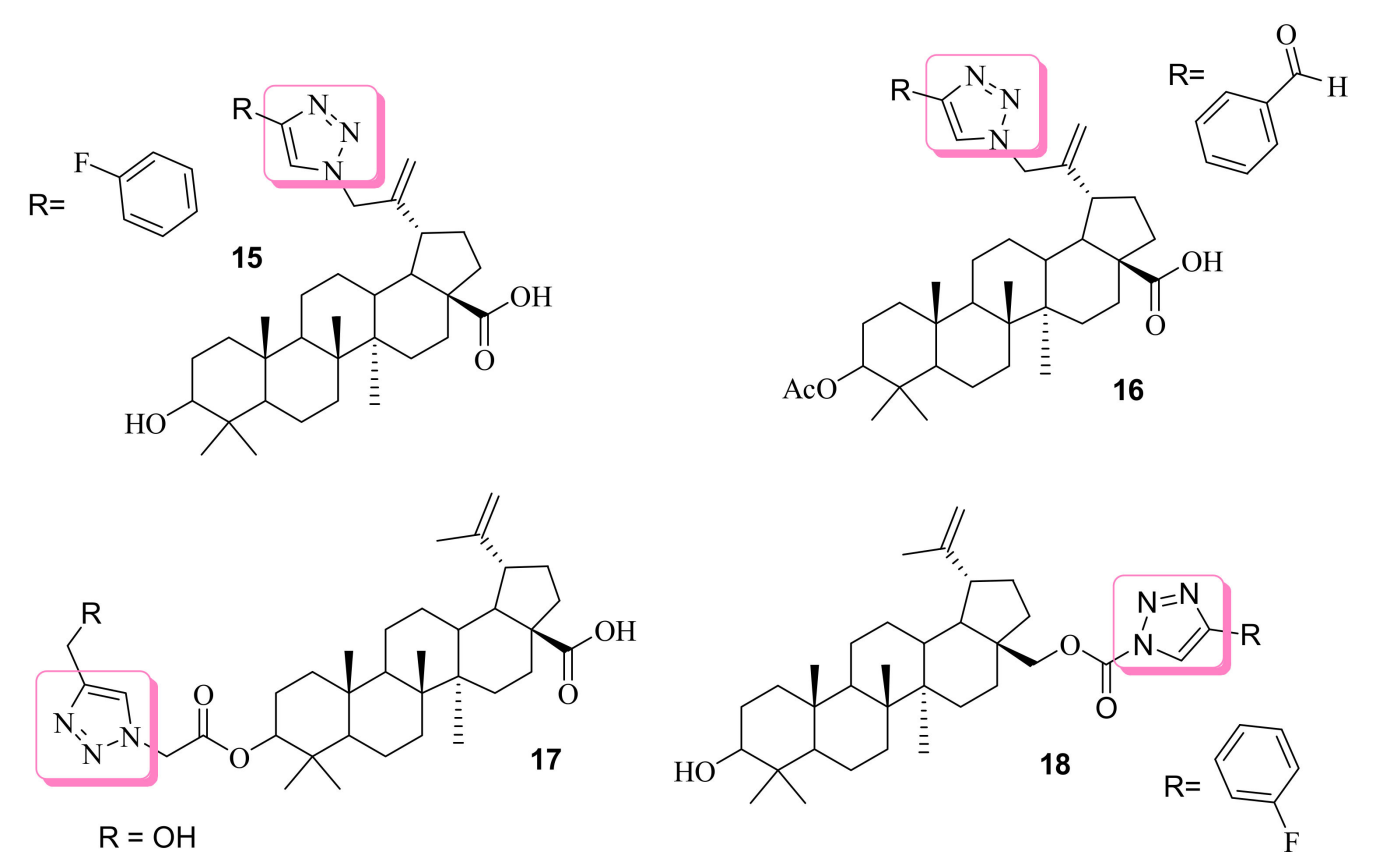
Betulinic acid-based 1,2,3-triazole analogues.

**Figure 3:**
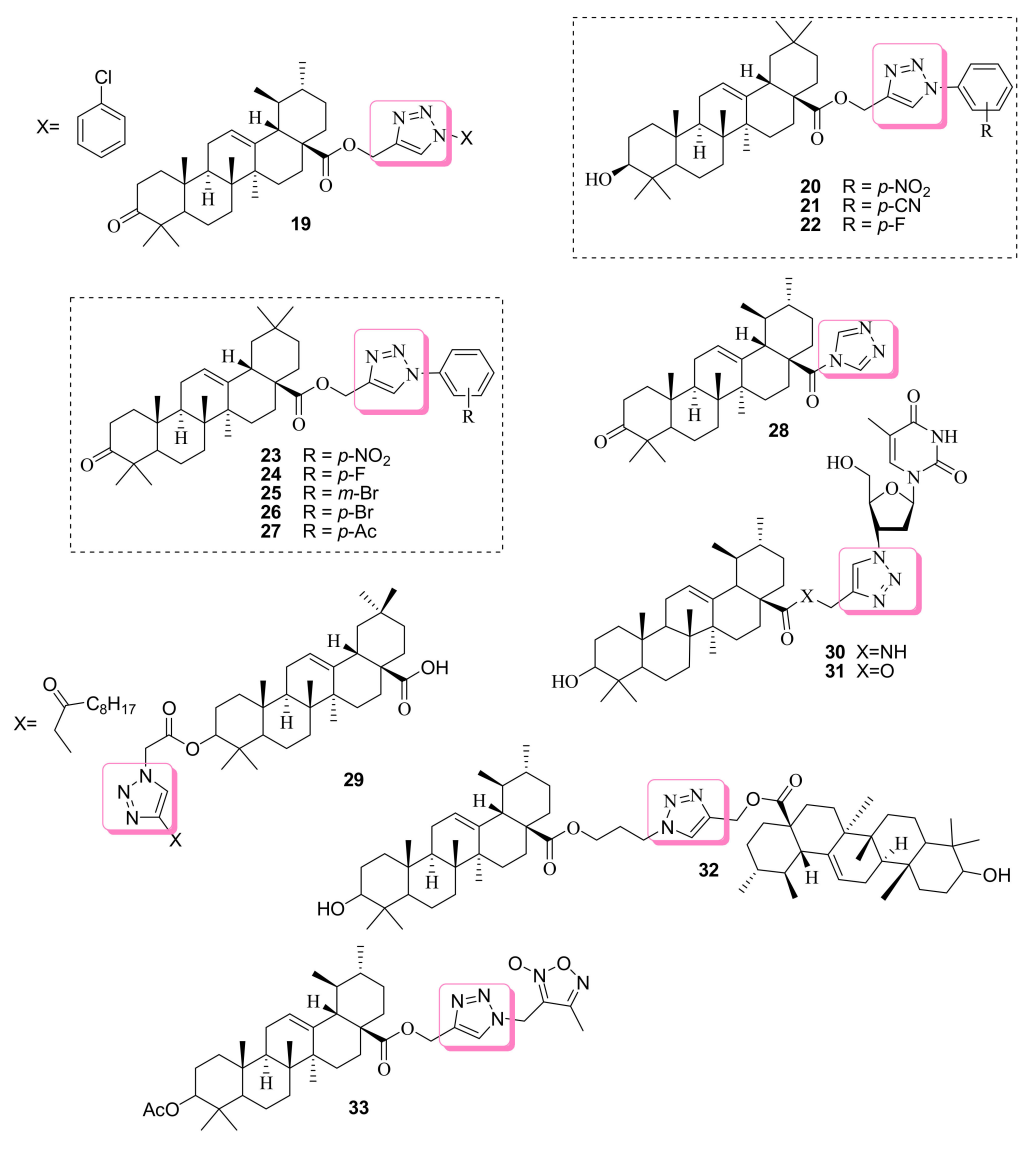

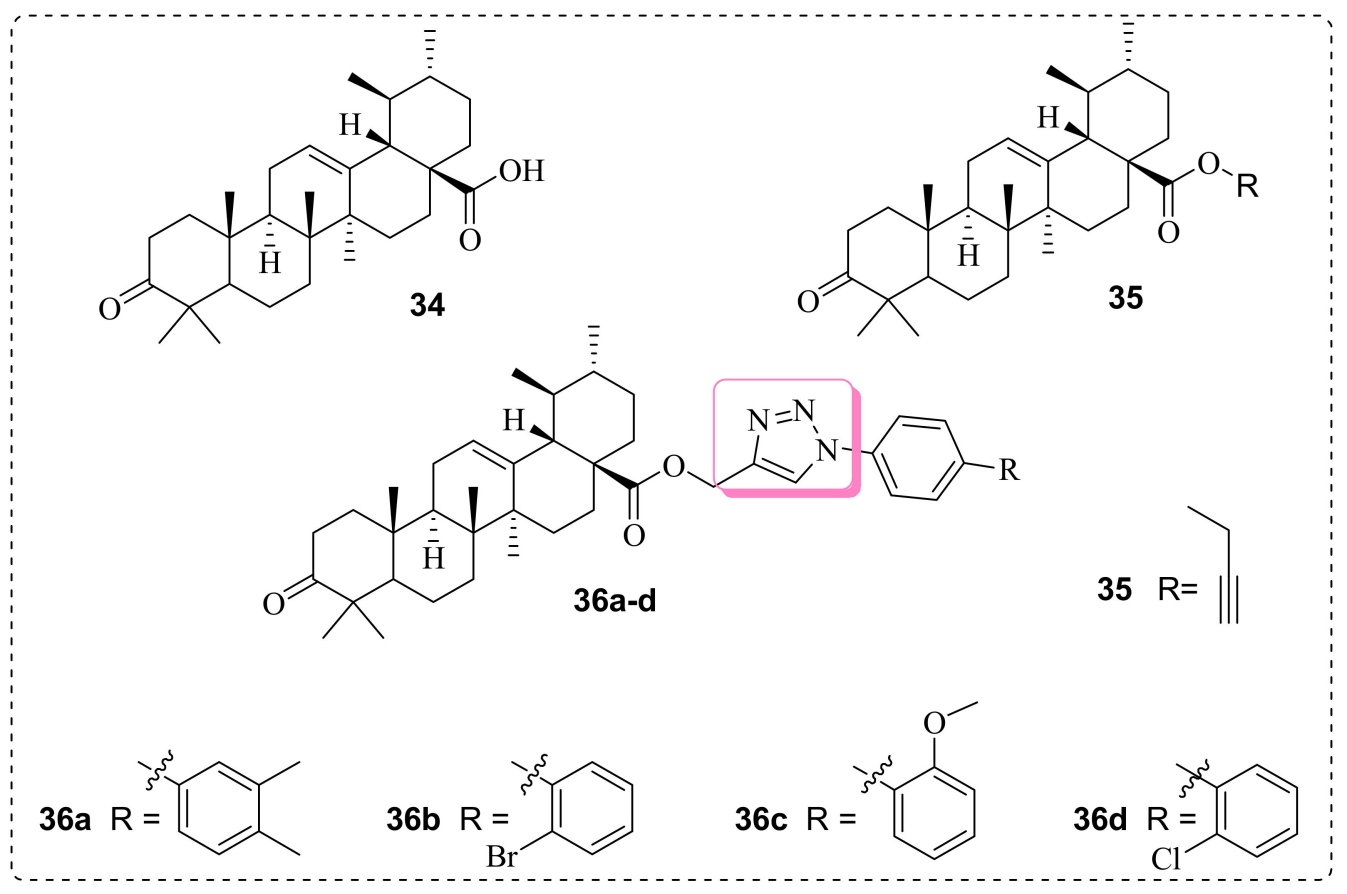
Ursolic acid and oleanolic acid-based triazole hybrid compounds.

A small library of novel amide-triazole-linked triterpenoid-AZT conjugates were prepared by Tuyet Anh Dang Thi and his group [59]. The anticancer effects of these hybrid compounds were evaluated against KB and Hep-G2 tumor cells. The most effective compounds that emerged from the in vitro cytotoxicity studies were (**5a**, **5b,** and **6** (Figure 2, Table 1)). With similar work, these authors also synthesized 12 novel ester-triazole-linked triterpenoid-AZT conjugates, and three compounds (**7**, **8**, and **9** (Figure 2, Table 1)) showed the most potent cytotoxicity activity against tumor cells (KB and Hep-G2) [60]. Majeed et al. [61] carried out the chemical transformation studies on betulinic acid through a concise synthesis of betulinic acid-based-1,2,3-triazole derivatives via click chemistry approach at C-3 position. Their cytotoxic effect was investigated against nine human tumor cell lines, namely THP-1, HL-60, DU-145, PC-3, HEP-2, MCF-7, A-549, SF-295, and HCT-15. Most of the derivatives exhibited higher cytotoxic profiles than the parent molecule. Most of the derivatives exhibited higher cytotoxic profiles than the parent molecule. In total, two compounds (i.e., **10** and **11** (Figure 2, Table 1)) showed impressive IC_50_ values (2.5 and 3.5 µM, respectively) against leukemia cell line HL-60 (5-7-fold higher potency than betulinic acid). The two compounds induced apoptosis, inhibited cell migration, and colony formation, mitochondrial membrane disruption followed by DNA fragmentation [61]. Khan et al. (2016) [62] synthesized a series of 1,2,3 triazole derivatives linked at C-3 and C-28 of betulinic acid using click chemistry approach. All the synthetic compounds were screened for their in vitro cytotoxicity against four human cancer cells, namely HL-60, PC-3, A549, and MiaPaCa-2. Among these derivatives, {1N (4-fluoro phenyl)-1H-1, 2, 3-triazol-4-yl} methyloxy betulinic ester (**12**) (Figure 2, Table 1) displayed better potency than the parent compound betulinic acid with IC_50_ values ranging from 5 to 7 µM. It disrupted the mitochondrial membrane potential, rendered Bcl-2 cleavage, Bax translocation, and decreased Bcl-2/Bax ratio. These events were accompanied by activation of caspases 9, 3, which cleaved the PARP-1. It also induced caspase-8, which is involved in the extrinsic apoptotic pathway. Therefore, it induces apoptosis through intrinsic and extrinsic pathways in human leukemia HL-60 cells [62]. Suman et al. (2017) [63] used click chemistry and Baylis–Hillman reaction protocols to synthesize betulinic acid-triazole derivatives. These derivatives were tested for their cytotoxic effects in human pancreatic cancer (MIA PaCa-2) and murine breast cancer (4T1) cell lines. Based on the in vitro assays, two compounds (**13** and **14**) (Figure 3, Table 1) were identified as lead compounds with IC_50_ values of 2.38 ± 0.45 µM (4T1), 1.36 ± 0.21 µM (MIA PaCa-2) and 2.62 ± 0.24 µM (4T1), 1.64 ± 0.20 µM (MIA PaCa-2) [63]. A series of triazole-linked betulin and betulinic acid analogs were synthesized via click chemistry at C-30 position by Shi and colleagues [64]. Their in vitro antitumor activities in leukemia cell line (HL-60) was studied. The in vitro cytotoxic analysis indicated that most of betulinic acid-based triazoles had higher cytotoxic activity compared to betulinic acid. In all the synthesized compounds, compound (**15**) (Figure 3, Table 1) showed the best IC_50_ value (1.3 µM) against leukemia cell-line HL-60, which had an eight- to nine-fold higher potency than betulinic acid [64]. Sidova et al. [65] used Huisgen 1,3-cycloaddition protocol to synthesize betulinic acid substituted triazole conjugates and evaluated them for in vitro cytotoxic activities against eight human cancer cell lines and two noncancer cell lines. Conjugates of 3β*-O*-acetylbetulinic acid were the most active compounds, and among them, compound **16** with triazole substituted by benzaldehyde was found to be the most active with an IC_50_ of 3.3 µM and therapeutic index (TI) of 9.1. The compound inhibited DNA and RNA synthesis and caused block in G0/G1 cell cycle phase which is highly similar to reference drug actinomycin D [65]. Chakraborty et al. (2015) [66] applied the azide-alkyne “click reaction” to synthesis a panel of novel betulinic acid analogues containing a triazole unit at C-3 attached through a linker. These analogues were screened for their anticancer activity against various cancer cell lines and normal human PBMC using MTT assays. Compound **17** (Figure 2, Table 1) showed the most potent inhibitory effect against cell line HT-29 with an IC_50_ value of 14.9 µM. They further investigated its mode of action and it exhibited much higher cytotoxicity than the standard drug, 5-fluorouracil but showed negligible cytotoxicity towards normal PBMC. The elevated level of ROS generation, activation of caspase 3 and caspase 9, DNA fragmentation, higher expression of Bax and Bad, lower expression of Bcl2 and Bcl-xl, and increased level of Bax/Bcl-xl ratio identified **17** (Figure 2, Table 1) as a promising inducer of apoptosis that follows a mitochondria-dependent pathway. Biophysical studies indicated that compound **17** (Figure 2, Table 1) acts as a minor groove binder to the DNA [66]. Novel triazole hybrids of betulin were synthesized using the 1,3-dipolar cycloaddition reaction between the alkyne derivatives of betulin and organic azides. In this study, bistriazole (compound **18**) (Figure 2, Table 1) was identified as a potent compound with an IC_50_ value of 0.05 µM against T47D (human ductal carcinoma cells) with 500-fold higher potency than cisplatin [67].

### 3.2. Anticancer Effects of Ursolic/oleanolic Acid-based 1,2,3-Triazole Molecules

Zhu et al. (2015) [68] investigated the anticancer effect of ursolic acid chlorophenyl triazole (UACT) (**19**) and 5-fluorouracil (5-FU) against human lung cancer cell lines (H209, 87-5 and Lu135) (Figure 3, Table 1). The results revealed a synergistic effect of the sequential treatment with UACT and 5-FU combination on cytotoxic activities, NF-kB protein activation, repression of TNF-induced NF-kB-dependent reporter gene expression, and TNF-induced COX-2, MMP-9, and Cyclin D1 activation in H209 cells. The synergism in apoptotic cell death was observed in H209, H69, 87-5, and Lu135 cells. The synergistic effect of UACT and 5-FU was observed at a concentration of 50 nM of UACT and 20 μM of 5-FU. Wei et al. [69] designed and synthesized a library of novel oleanolic acid coupled 1,2,3-triazole derivatives using a Cu(I) catalyzed Huisgen 1,3-dipolar cycloaddition reaction. The antiproliferative analysis revealed some of the synthetic compounds excellent anticancer activity on human tumor cell lines such as HeLa, HepG2, HCT116, A375-S2, and HT1080. Among all the derivatives, compounds **20**, **21**, and **22**, with *p*-NO_2_, *p*-CN, and *p*-F substitutions at an aromatic ring (Figure 3, Table 1) possessed the best inhibitory activity against HT1080 cells. The pharmacology experiments showed that compound **20** significantly induced HT1080 cell apoptosis [69]. In a similar study, Fengran et al. [70] further synthesized 15 novel 3-oxo-oleanolic acid coupled 1,2,3-triazole derivatives. Compounds **23**-**27** (Figure 3, Table 1) displayed the best potent activity and selectivity against A375-S2 and HT1080 cell lines with IC_50_ values ranging from 1.69-2.82 µM. In this study, it was evident that the 3-oxo OA derivatives displayed better anticancer activity compared to OA derivatives [70]. 

**Figure 4:**
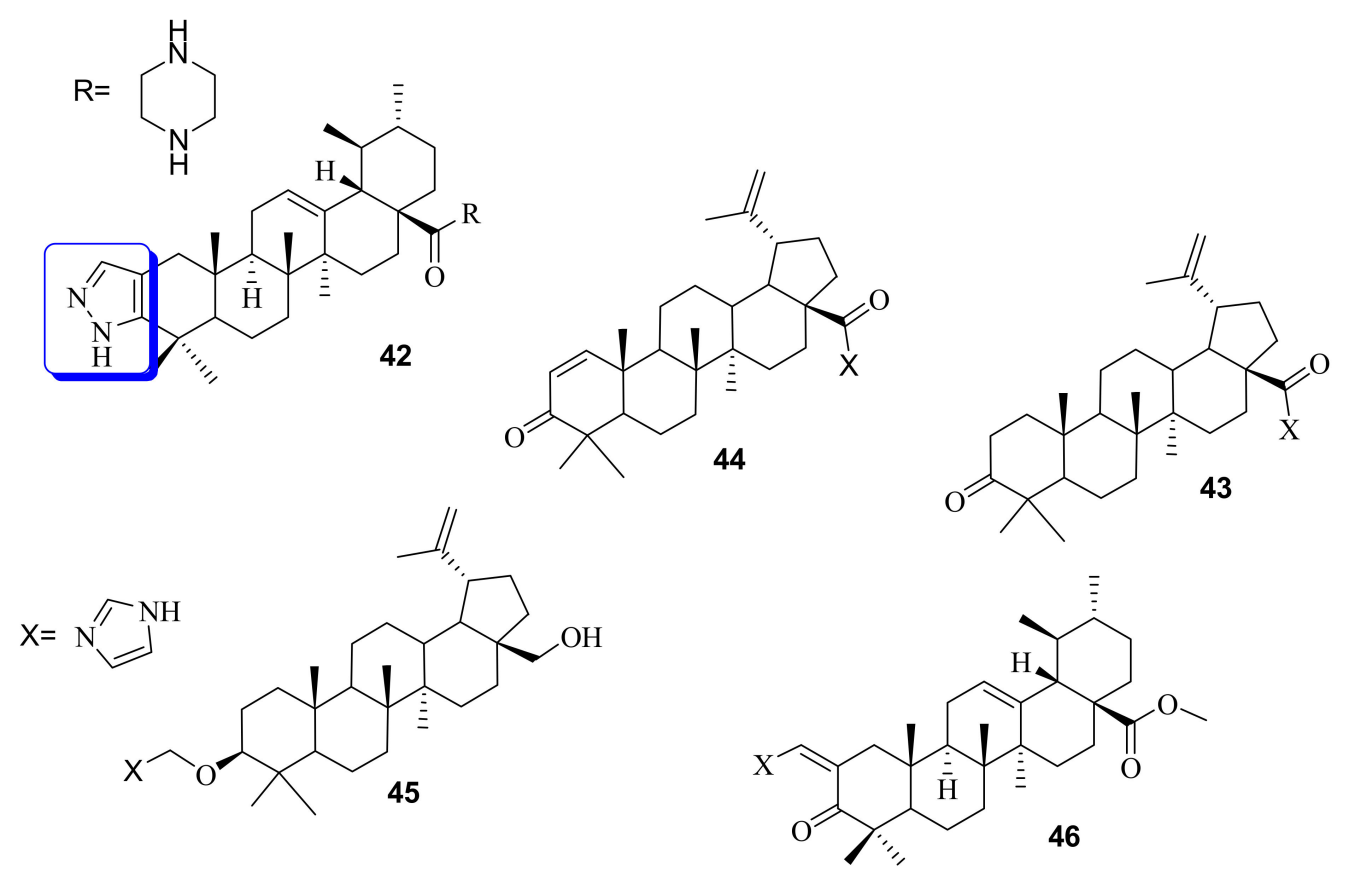
Ursolic acid and betulinic acid-base pyrazole derivatives.

Leal and colleagues [71] developed a series of novel N-acylimidazoles and N-alkylimidazoles ursolic acid analogues bearing imidazole or triazole in different positions of the ursane skeleton and evaluated the anti-proliferative effect in pancreatic cancer cells (AsPC-1). The tested compounds exhibited improved the antiproliferative activity against AsPC-1 cells than the parent compound, ursolic acid. Compound **28** (Figure 3, Table 1) was seven-fold more potent than ursolic acid with an IC_50_ value of 1.9 µM. Compound **28** also indicated the induction of p21^waf1^, p53, and NOXA, which led to cell cycle arrest and AsPC-1 apoptosis [71]. A total of 18 derivatives of oleanolic acid were prepared and investigated for their in vitro cytotoxic activity by Pertino et al. [72], and compound **29** (Figure 3, Table 1) showed better activity with an IC_50_ value of 8.9 µM against gastric epithelial adenocarcinoma cells (AGS cells). Ursolic acid derivatives containing either an amide-triazole-AZT linkage (as in **30**) or an ester-triazole-AZT linkage (as in **31**) (Figure 4, Table 1) showed moderate cytotoxic activity [59]. High cytotoxicity was observed for ursolic acid derivative, **32** (Figure 3, Table 1) modified at position C-28. This compound GI_50_ values was 1.4 µM against the human breast tumor cell lines (MDA-MB-231) [73,74]. Ursolic acid derivatives (**33**) (Figure 3, Table 1) containing heterocyclic fragments such as 1,2,3-triazole and 3-(methyl)-4-methyl-1,2,5-oxadiazole-2-oxide linked at position C-28 displayed the best cytotoxic activity against MCF-7 cells with IC_50_ value of 1.55 ± 0.08 µM comparable to doxorubicin. The molecular docking studies indicated that the most likely cytotoxic mechanism of action is the affinity of hybrid **33** to Mdm2 binding sites [75].

A number of ursolic acid-1-phenyl-1H-[1,2,3]triazol-4-ylmethylester congeners were designed and synthesized by Rashid et al. (2013) [76] in an attempt to develop potent antitumor agents. To synthesize these congeners, UA was subjected to oxidation using Jones reagent at 0 °C that resulted in the formation of C-3 oxidized derivative **(34)**(Figure 3) in almost quantitative yield. Propargylation of the carboxylic group at C-28 was tried with different bases, including NaHCO_3_, Cs_2_CO_3_, pyridine, Et_3_N, and DBU. Cs_2_CO_3_ condition in dry THF delivered the well-poised alkyne derivative **35** (Figure 3) in excellent yield. On the other hand, aromatic azides were prepared from their respective anilines by diazotization with sodium nitrite in acidic conditions followed by displacement with sodium azide in good to excellent yield. 1,3-dipolar cycloaddition reaction of **35** with aromatic azides in the presence of CuSO_4_.5H_2_O and sodium ascorbate in t-BuOH: H_2_O (2:1) resulted in the formation of 1,4-substituted-triazolyl derivatives **36a**–**d** (Figure 3) in excellent yields. All the compounds were evaluated for anticancer activity against a panel of four human cancer cell lines, such as A-549 (lung), MCF-7 (breast), HCT-116 (colon), THP-1 (leukemia), and a normal human epithelial cell line (FR-2) using sulforhodamine-B assay. The pharmacological results showed that most of the compounds displayed a high level of antitumor activities against the tested cancer cell lines compared with ursolic acid. Compounds **36a**–**d** (Table 1) were found to be the most potent compounds.

## 4. Pyrazole

Pyrazole, a five-membered heterocyclic ring, is the most studied compound among the azole family due to its interesting pharmacological properties such as antibacterial, anticancer, antioxidant, antifungal, antidepressant, antituberculosis, anti-inflammatory, and antiviral activities [77,78,79]. The pyrazole ring is the best scaffold for synthesizing various pharmaceutical compounds with different therapeutic activities and good safety profiles [80]. Most of the synthesized pyrazole derivatives have been studied for their in vitro antiproliferative activities and in vivo antitumor activity, often resulting in promising lead scaffolds [81,82,83,84,85]. Recently, Bennani et al. also demonstrated that pyrazole derivatives possess high antiproliferative activity against tumors cells such as breast (MCF-7), lung (A-549), liver (HepG-2), and brain (HeLa) cell lines [86]. In a continuing interest in pyrazole-based derivatives, PTs with pyrazole rings have been reported.

### Anticancer Effects of Triterpenoid-based Pyrole Molecules

**Scheme 1:**
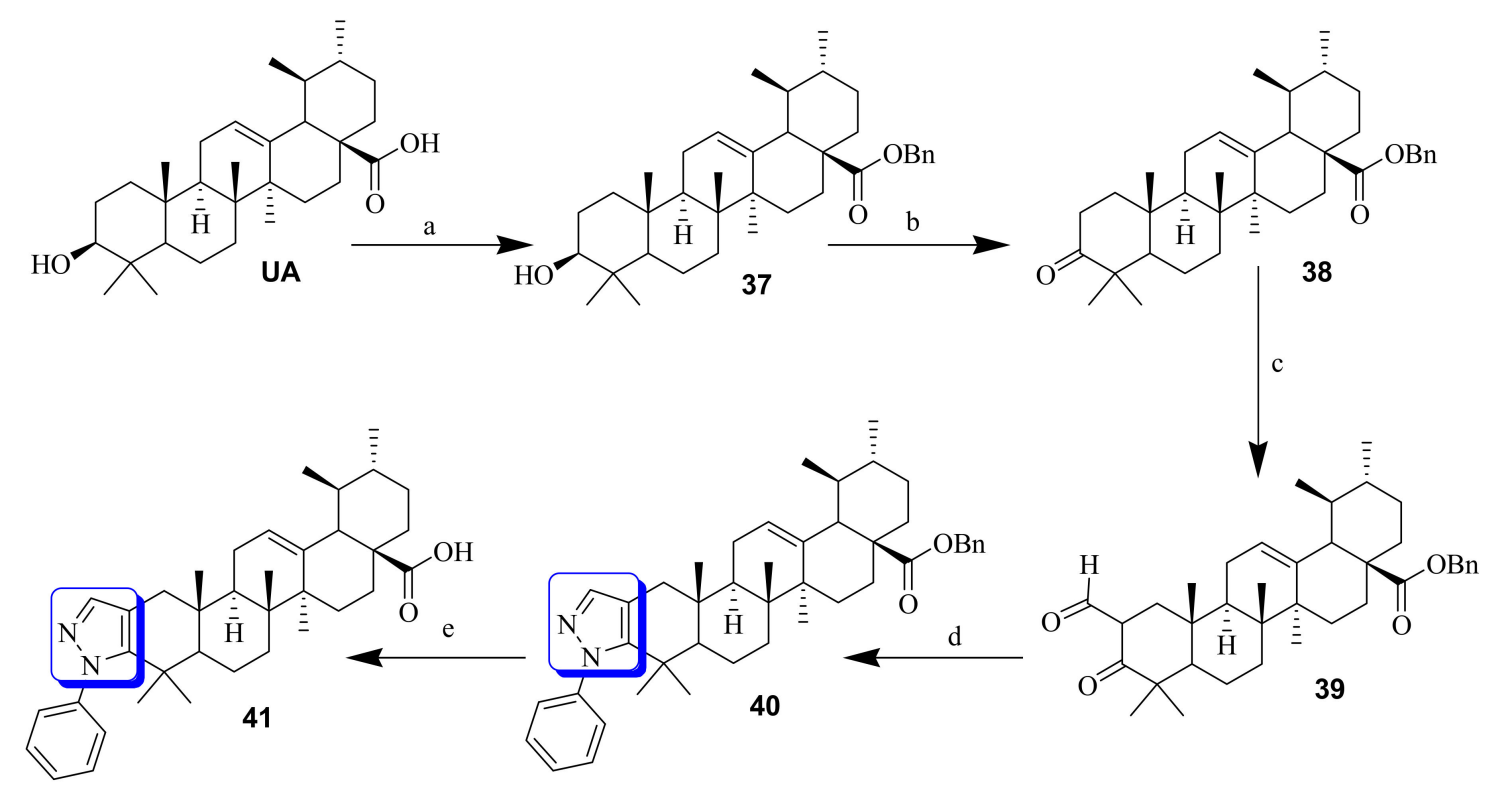
Reagents and conditions: (a) BnBr, K_2_CO_3_, DMF, 60 °C, 92%. (b) PCC, CH_2_Cl_2_, 0 °C to rt, 84%. (c) R_1_CO_2_Et (R_1_ = H, Me), NaOMe, THF, rt; R1 CO2Et (R_1_ = CF_3_), t-BuOK, THF, rt, 70%. (d) R_2_ NHNH_2_·HCl, EtOH, rt. (e) H2, Pd/C, MeOH, rt.

Sun et al. [87] synthesized pyrazole-fused ursolic acid derivative **41** (Scheme 1**, Table 2**) and evaluated their in vitro anticancer activities against tumor cells, such as cervical carcinoma (HeLa cells), hepatocellular carcinoma (HepG2 cells), fibrosarcoma (HT1080 cells), mammary adenocarcinoma (MCF-7 cells), and neuroblastoma (SK-N-MC cells). This compound induced apoptosis by hyperstimulation of macropinocytosis and caused the accumulation of vacuoles derived from macropinosomes based on transmission electron microscopy, time-lapse microscopy, and labelling with extracellular fluid phase tracers.

Li and colleagues [88] synthesized novel ursolic acid derivative **42** (Figure 4, Table 2) with a nitrogen-containing heterocyclic scaffold and the privileged fragment at the C-28 position by treating UA with acetic anhydride (Ac_2_O) in dry pyridine under the 4-dimethylaminopyridine (CH_3_)_2_NC_5_H**_4_**N) at room temperature for 2 h. The 3-acetylated UA was treated with oxalyl chloride at room temperature for 3 h to produce an intermediary 28-acyl chloride. This compound was then mixed at room temperature for 2 h to synthesize compound **42**. These authors investigated cell proliferation, apoptosis induction, and cell cycle in human breast cancer lines. The results indicated that **42** significantly increased the number of SUM149PT and HCC1937 cells lines in the G_0_/G_1_ phase in a dose-dependent manner. Compound **42** significantly induced apoptosis in breast cancer more than UA [88]. Chen et al. [89] also synthesized **42**, which exhibited a remarkable growth of the inhibitory effect against HL-60 cells leukemia cells with an IC_50_ value of 0.91 µM, approximately 100-fold more potent than UA [89].

Santos et al. [90] semi-synthesized novel BA derivatives **(43, 44,** and **45)** (Figure 4, Table 2) with 18–45 times improved cytotoxic activity against HepG2 cell lines and induced apoptosis and cell cycle arrest in HepG2, HeLa, and Jurkat cell lines. The mechanism of apoptosis induction was mediated by the activation of the post mitochondrial caspases-9 and -3 cascade and possibly by mitochondrial amplification loop involving caspase-8. Among these compounds, **43** is the most active compound with an IC_50_ value 45 times lower than BA on HepG2 cells and 61 times lower than those found for the non-tumoral Chang liver cells [90]. Leal et al. [91] synthesized a series of novel oleanane derivatives bearing imidazole carbamates, N-alkylimidazoles or N-acylimidazoles and evaluated their antiproliferative activity against AsPC-1 pancreatic cancer cell lines. The results revealed that the N-alkylimidazole **46** (Figure 4, Table 2) was the most active compound with apoptosis induction abilities correlated with upregulation of NOXA and downregulation of Bcl-xL. The antiproliferative activity of compound **46** was further tested in more solid tumor cell lines with IC_50_ values lower than 1 μM.

## 5. Indole

Indole ring is one of the nitrogen heterocyclic compounds widely found in nature and commonly found in plant hormones, including tryptophan and auxins, serotonin or 5-hydroxytryptamine, and melatonin, which plays a significant role in the animal physiological and biochemical process [92]. The hybridization of indole with PTs is an effective strategy to search and develop novel anticancer candidates. The resulting single molecule containing one or more pharmacophores with different mechanisms of action may lead to the enhancement of the desired properties of the combined components and potentially reduce the drug resistance.

Anticancer Effects of Triterpenoid-based Indole Molecules

**Scheme 2:**
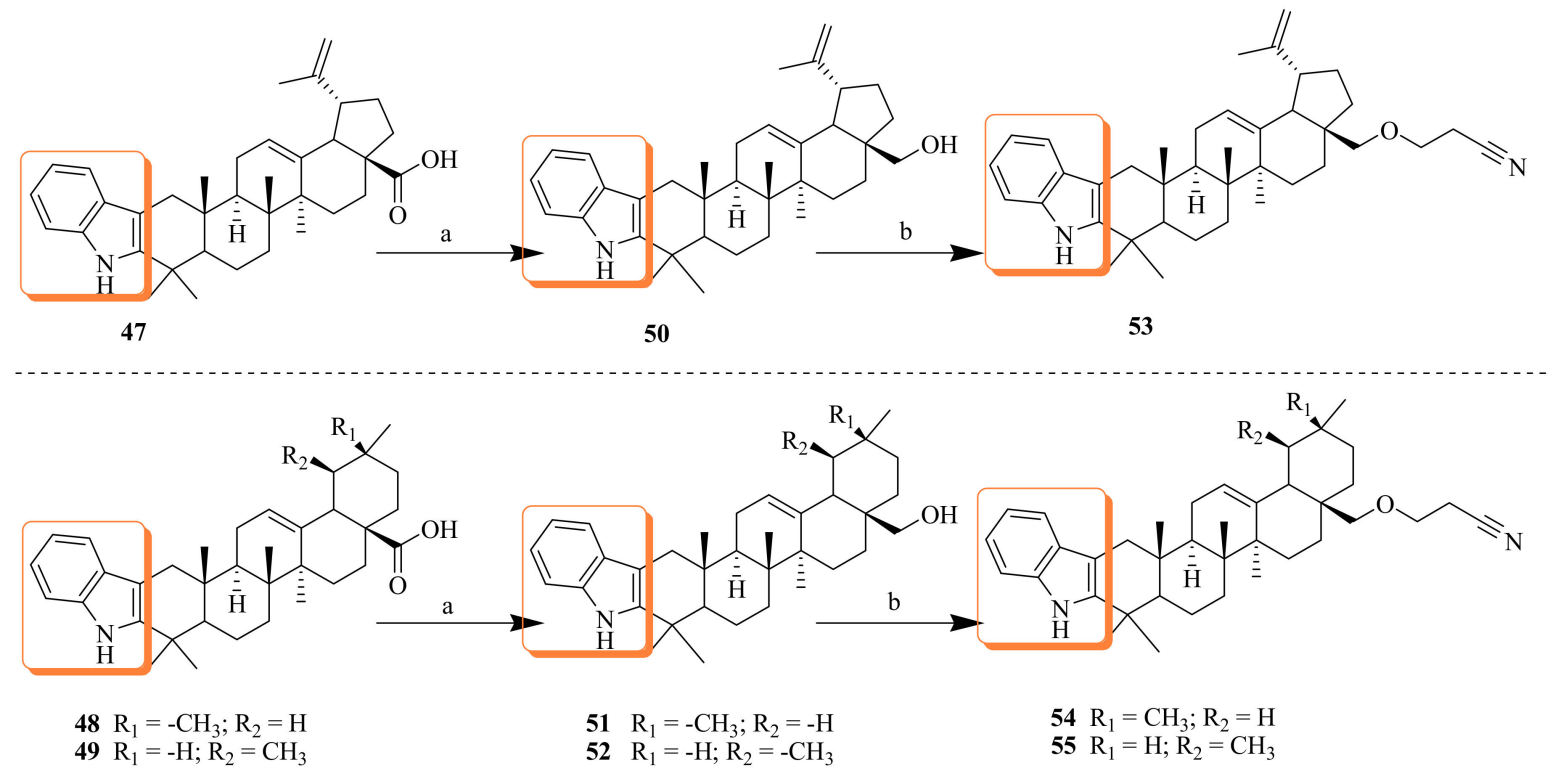
Conditions: (a), LiAlH4, THF, 2 h; (b), CH2=CHCN, 30% KOH, TEBAC, 1,4-dioxane, 5 h.

Khusnutdinova et al. prepared novel 2,3-Indolotriterpenic alcohols by successive modification of 3-oxo triterpenic acids (Fisher reaction, reduction of C17-COOH, cyanoethylation) (Scheme 2) and evaluated them for in vitro antitumor activity. Compounds 2,3-indolouvaol (**51**) and 2,3-indolo-28-cyanoethoxybetulin (**53**) showed potential in vitro antitumor activity against NCI-H522 and COLO 205 cells, inducing the death of 12.65% and 42.78% of these cells, respectively [93].

**Figure 5:**
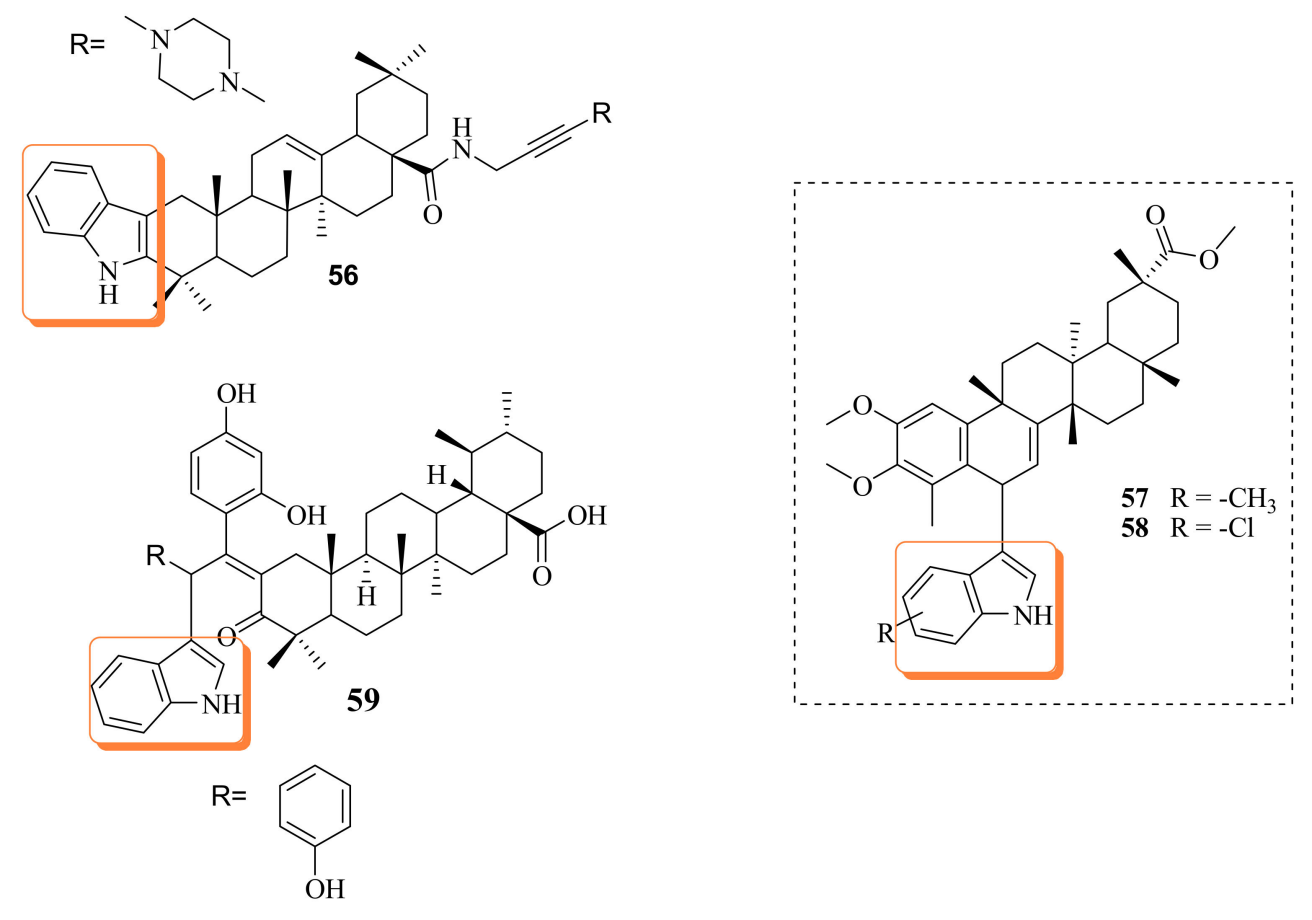
Oleanolic and ursolic acid-based indole hybrids.

Khusnutdinova et al. [94] attempted the modification of the carboxyl group of [3,2b]-indolotriterpenic acids (N-propargylation, Cu(I) catalyzed Mannich reaction) with N-methylpiperazine and morpholine moiety. Among the modified compounds, oleanane-type conjugate with N-methylpiperazine **56** (Figure 5) displayed the highest anticancer potency against leukemia cell line SR (‒2.2%) and nonsmall cell lung cancer cell line NCI-H460 (‒25.3%). Tang et al. [95] designed and synthesized a series of novel oleanolic acid-based C(6)-indole substituted celastrol analogues. Among all these semisynthetic analogues, compounds **57** and **58** (Figure 5) displayed excellent in vitro antiproliferative activities against Bel7402 cancer cells with IC_50_ values of 0.02 µM and 0.01 µM, respectively [95]. Fan et al. [96] synthesized a series of ursolic acid-based indole derivatives with compound **59** (p< 0.05) (Figure 5), showing promising therapeutic effects against the growth of U251 and C6 glioma cells at a concentration of 10 μM. This compound inhibited glioma cell development, induced apoptosis, and cell cycle arrest in the G0/G1 phase with decreasing population in the G2/M and S phases via the down-regulation metabolic pathways. This compound is a potential anticancer agent for the treatment of glioma [96].

## 6. Piperazine

**Figure 6:**
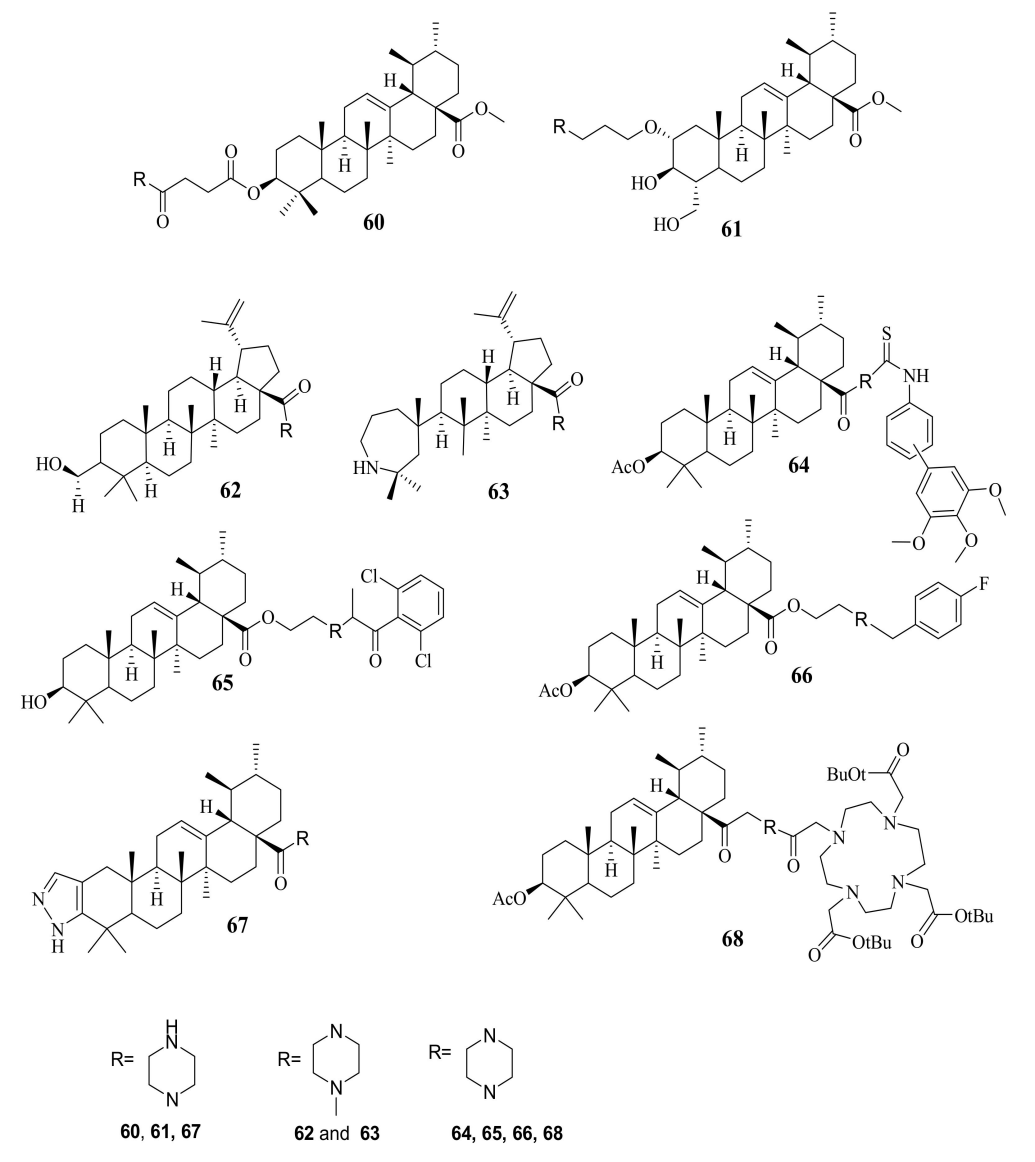
Ursolic acid and betulinic acid-based indole hybrids

The piperazine is a nitrogen-containing heterocyclic ring known for its medicinal importance. It is considered the most attractive scaffold for the development of novel anticancer agents [97]. In 2016, Rathi and co-workers [98] published a review of the anticancer potential of piperazine derivatives. Molecular hybridization has been identified as one of the successful strategies for developing novel chemotherapeutic candidates involving the combination of two or more different bioactive fragments. The combination of PTs and piperazine into a single-molecule (hybrid) has been reported with potential anticancer activity. Compounds methyl 3-O-[4-(1-piperazinyl)-4-oxo-butyryl]olean-12-ene-28-oate (**60)** and compound 2-O-[4-(1-piperazinyl)-4-oxo-butyryl]-3,23-dihydroxyurs-12-ene-28-oate (**61)** were synthesized by Zhao et al. [99]. These compounds were evaluated for their biological activity using the Cell Counting Kit-8 method, and Western blotting analysis on A549 cells, MCF-7cells, and Hela cells. Both compounds **60** and **61** exhibited excellent anticancer activity than the reference drug, Gefitini [99]. Giniyatullina et al. [100] synthesized A-azepanobetulinic acid *N*-methylpiperazinylamide through a series of transformations (oximation, Beckmann, reduction) of *N*-methylpiperazinylamide. They evaluated their in vitro cytotoxicity against some cell lines, namely LNCAP, PC3 22RW1, Huh7, and VA13. Compounds **62** and **63** (Figure 6, Table 3) displayed the most potent cytotoxicity against the five cancer cell lines with an IC_50_ ranging from 0.37 µM to 3.58 µM [100]. Compound **64** (Figure 6, Table 3) effectively induced cell apoptosis in HepG2 cells [101]. 

**Figure 7:**
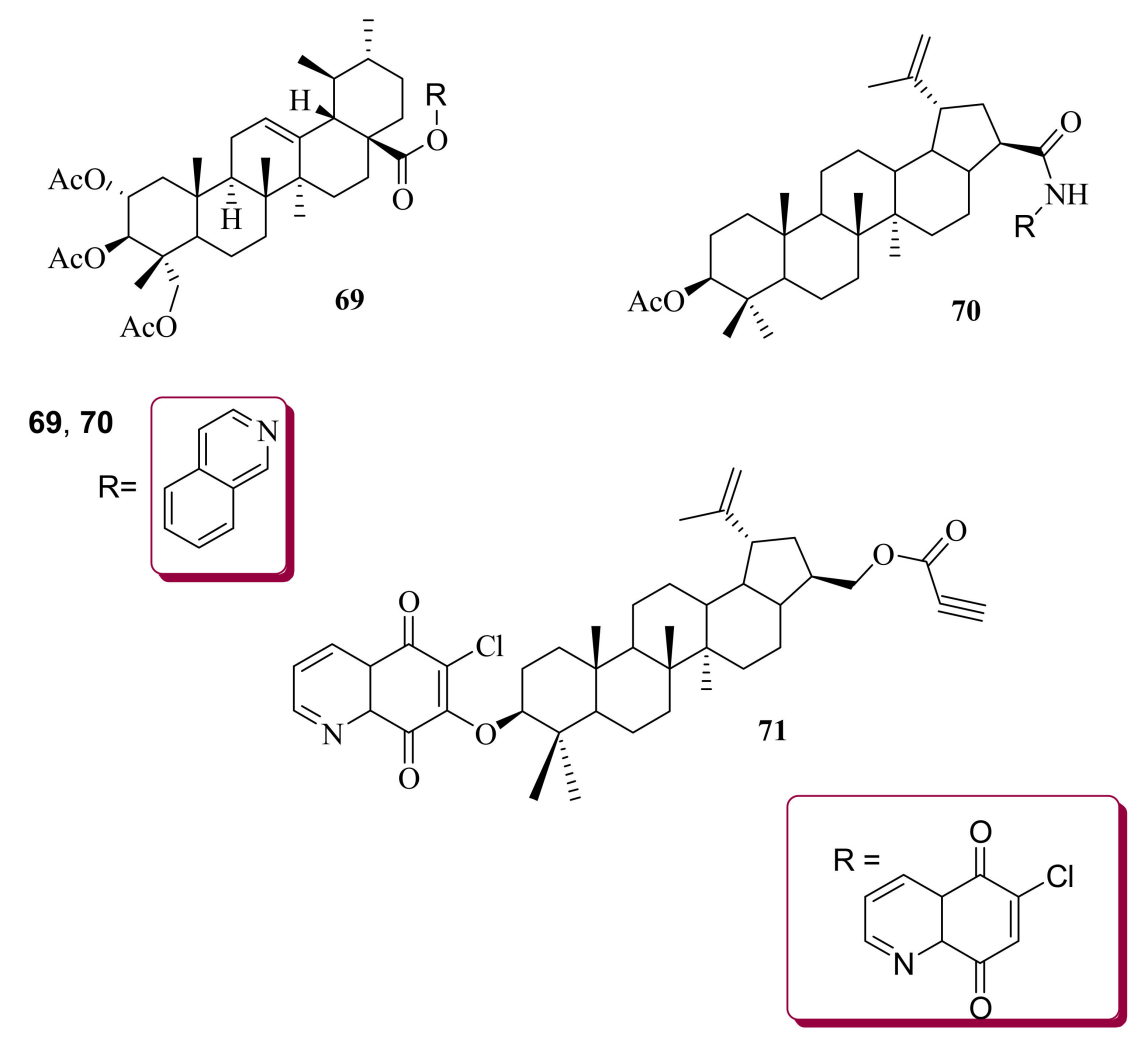
Ursolic acid and betulinic triterpenic analogues with amminoquinoline heterocyclic hybrid scaffold.

The ursolic acid derivative **65** with an acyl piperazine moiety at C-28 was synthesized by treating ursolic acid with ethylene dibromide (C_2_H_4_Br_2_) in Dimethylformamide (DMF) for 24 h. The obtained compound was introduced with piperazine in DMF in the presence of Potassium carbonate (K_2_CO_3_) at 80 ˚C, and then reacted with an aromatic to obtain the targeted compound. The newly synthesized compound was evaluated for anticancer activity against human gastric cancer (MGC-803) and breast cancer (Bcap-37) cell lines using standard MTT assay in vitro. The compound showed a cell apoptosis-inducing effect higher than the positive control, HCPT, and UA [102]. Wang et al. [103] designed and synthesized 19 new nitrogen heterocycle-containing ursolic acid derivatives and evaluated their potential antiproliferative activity against the cervical (Hela) and gastric (MKN45) cell lines. The potent anticancer compound was found to be compound **66** (Figure 7, Table 3) with IC_50_ values of 2.6 µM and 2.1 µM. The study of the mechanism and the in vivo antitumor study of this compound demonstrated reduced apoptosis regulator (Bcl-2/Bax) ratio, disrupted mitochondrial potential and induced apoptosis, and suppressed the growth of Hela xenografts in nude mice [103]. Li et al. [88] indicated that apoptosis in breast cancer cells was induced by ursolic acid piperazine hybrid derivative (compound **67** (Figure 6)), along with cell cycle arrest induction at S and G0/G1 phase. Thus, compound **67** is a promising therapeutic agent for the treatment of breast cancer. With earlier literature report by Chen et al. [89] suggesting the therapeutic potential of a similar compound against leukemia as summarized in Table 3 [89]. Kahnt et al. [104] prepared 11 conjugates containing piperizine hybrid heterocycles. Amongst all the ursolic acid derivatives, compound **68** was most potent with EC_50_ values of 1.5 µM for A375 melanoma and 1.7 µM for A2780 ovarian carcinoma) [104].

## 7. Aminoquinolines

The principle of molecular hybridization is efficient as it is centered on the combination of two different distinct pharmacophores by linking covalently two or more structural entities with distinct biological functions. The positive synergistic effects of these compounds can also occur along with expected pharmacokinetic and pharmacodynamic profiles. In addition, by enhancing bioavailability and distribution through cell membranes of cell organelles, this process can solve a variety of common problems associated with drug molecules and shielding active compounds from enzyme degradation [105]. Quinones are a large and substantial family of cyclic compounds combined with a large variety of medicinal agents for therapeutic applications [106]. Figure 7 represents ursolic acid and betulinic triterpenic hybrids (**69**–**71**) with aminoquinoline heterocyclic scaffold. Sommerwerk et al. [107] synthesized a series of pentacyclic triterpenic derivatives and tested their activity against a number of human cancer cells using SRB assay to access their cytotoxicity. A triacetylated 4-isoquinolinyl derived compound of asiatic acid (**69)** was the most promising compound with IC_50_ values of 0.19 µM for melanoma (518A2), 0.22 µM for colorectal adenocarcinoma (HT29), 0.54 µM for breast (MCF7), 0.29 µM for lung adenocarcinoma (A549), 0.08 µM for ovarian (A2780) and 3.23 µM nonmalignant mouse fibroblast (NiH3T3). This compound was further examined by apoptosis through propidium iodide/acridine orange staining, fluorescence spectroscopy, and cell cycle investigation [107]. Hoenke et al. [108] synthesized a sequence of betulinic acid amide derivatives in an attempt to investigate their activity against some cancer cell lines compared to NiH3T3. Compound **70** was the most cytotoxic and therefore showed lower EC_50_ values of 1.48 ± 0.1 µM for melanoma (A375), 4.65 ± 0.5 µM (MCF7), 2.16 ± 0.2 µM (A2780), 2.26 ± 0.2 µM for hypopharyngeal carcinoma (FaDu) and > 30 µM (HT29) compared to > 91.2 µM (NiH3T3). On the other hand, doxorubicin was used as a reference drug with EC_50_ values of > 30 µM (HT29), 1.1 ± 0.3 µM (MCF7), 0.01 ± 0.01 µM (A2780), not detected for A375 and FaDu compared to 1.3 ± 0.6 µM (NiH3T3). Therefore compound **62** can be further investigated as a antitumor agent [108]. Kadela-Tomanek et al. [109] synthesized Betulin-1,4-quinone hybrids using a linker method of combining two active structures in an attempt to produce a more biologically active derivatives with anticancer activity and better bioavailability. The synthesized derivatives were tested in vitro against a number of human cancer cells such as breast (MCF-7, T47D, and MDA-MB-231), melanoma (C-32), lung (A549), colon (Colo-8), and gliblastoma (SNB-19). They found out that their derivatives were cytotoxic towards the cancer cells with NAD[P[H-quinone oxidoreductase (NQO1) protein level namely MC-7, C-32 and A549. Compound **71** was the most promising hybrid with IC_50_ values of 1.27 ± 0.06 µM (C-32), 2.43 ± 0.68 µM (MDA-MB-231), 6.67 ± 1.30 µM (Colo-8), 14.9 ± 2.30 µM (MCF-7). However, its cytotoxicity against SNB-19 and T47D was not impressive with IC_50_ values of 41.99 ± 0.11 µM and 99.39 ± 6.98 µM, respectively. Compound **71** reduced the proliferative activity of all tested cells during enzymatic mechanism of action. This compound was tested for its transcriptional activity of gene encoding of cell-cycle (p21 and p53), proliferation maker (H3-histone) and apoptosis. Furthermore, this compound interacted with hydrophobic matrix of the active site of the enzyme near FAD, Trp105, Phe178, and Tyr128 cofactor, which was observed by an increase in gene expression (TP53) [109].

## 8. Perspectives and Concluding Remarks

The growing incidences of cancer are one of the major issues in the developing world. Novel therapeutic agents that would be useful for the treatment of cancers that have acquired resistance to the existing drugs are desperately needed. PTs have the advantage of creating various derivatives with potential pharmacological properties. This review summarized the synthesized PTs derivatives with fused nitrogen-containing heterocyclic scaffolds, which have displayed significant anticancer activity. We noted that recent studies had been done on triterpenoid with nitrogen-containing heterocyclic scaffolds to understand the mechanism of action of these hybrids. The introduction of various nitrogen-containing heterocyclic moieties in PTs brings new mechanism of actions that lead to high anticancer activity. The hybrid compounds exhibited high anticancer activity such as **18**, (**36a-36d**), **43**, **44**, **45**, **46**, and **68**. Some hybrid compounds have been reported to be the modulators for multiple targets at different stages of cancer progression, such as proliferation, angiogenesis, metastasis, and apoptosis. Almost all the reported hybrid compounds displayed enhanced anticancer activity compared to the parent PTs. The above-mentioned significant points identify the enormous potential of these triterpenoid-based nitrogen-containing heterocyclic moieties in pharmaceutical applications suggesting a massive scope for these promising compounds because of their diverse molecular targets.

## Figures and Tables

**Table 1:** Mechanism and cytotoxicity of pentacyclic triterpenoids (PTs)-based triazole scaffolds on different cancer cells.

Hybrid Compounds	Mechanism/Pathways	Cancer Cell Lines	Hybrid Compounds (IC_50_ µM)		Reference Molecules (IC_50_ µM)		Bibliography
**5a**	n.r	KB Hep-G2	(8.1) (6.6)	Betulinic acid (27.5) (23.9)		Ellipticine (1.2) (1.2)	[59]
**5b**			(4.6) (3.5)				
**6**			(8.1) (5.9)				
**7**			(5.9) (7.0)				[60]
**8**			(6.3) (7.4)				
**9**			(7.7) (11.2)				
**10**	Induced apoptosis and inhibit cell migration, disruption of mitochondrial membrane.	THP-1 HL-60	(4.5) (2.5)	Betulinic acid (20) (17)		------	[61]
**11**			(8) (3.5)				
**12**	Induced apoptosis through both intrinsic and extrinsic pathways.	HL-60 MiaPaCa-2 PC-43 A549	(7) (5) (7) (7)	Betulinic acid (8) (7) (7) (8)		------	[62]
**13**	n.r	4T1 MIAPaCa-2	(2.38 ± 0.45) (1.36 ± 0.21)	Betulinic acid (6.29 ± 0.96) (25.63 ± 3.79)		------	[63]
**14**			(2.62 ± 0.24) (1.64 ± 0.20)				
**15**	nr	HL-60	(1.3 ± 0.1)	Betulinic acid 11.5 ± 2.8		------	[64]
**16**	Inhibit DNA and RNA, cause block in G0/G1 cell cycle phase similar to actinomycin D.	CCRF-CEM CEM-DNR K562 K562-TAX A549 HCT116 HCT116p53^-/-^ U2OS	(3.3) (4.0) (3.6) (3.9) (14.8) (6.4) (9.5) (12.8)	Betulinic acid (45.5) (45.4) (40.0) (43.1) (43.4) (38.0) (>50.0) (>50.0)		------	[65]
**17**	Induce apoptosis, acts as a minor groove binder to the DNA	HT29	(14.9 ± 1.3)	Betulinic acid (14.9 ± 1.3)		------	[66]
**18**	n.r	T47D2 MCF-7 SNB-19	(0.05 ± 0.01) (0.09 ± 0.01) (0.08 ± 0.01)	Betulinic acid (neg) (17.7 ± 1.2) (neg)		Cisplatin (4.9 ± 1.1) (5.5 ± 1.0) (2.3 ± 0.05)	[67]
**19**	Activate NF-kB protein, repression of TNF-induced NF-kB-dependent reporter gene expression, and TNF-induced COX-2, MMP-9 and Cyclin D1 activation	n.r	n.r	n.r		n.r	[68]
**20**	Apoptosis inducer in HT1080 cells	HeLa HepG2 HCT116 A375-S2 HT1080	(10.85) (24.15) (12.28) (4.97) (3.51)	Oleanolic acid (>200) (>200) (>200) (>200) (>200)		5-Fu (26.18) (67.64) (35.16) (90.74) (25.46)	[69]
**23**	n.r	A375-S2 HT1080	(2.82±0.11) (1.69±0.26)	Taxol (59.57±0.17) (35.18±0.44)		5-Fu (59.57±0.17) (35.18±0.44)	[70]
**24**			(36.54±0.39) (1.86±0.17)				
**25**			(13.98±0.78) (1.73±0.45)				
**26**			(34.87±0.62) (1.82±0.16)				
**27**			(4.76±0.11) (1.84±0.16)				
**28**	Induction of p21^waf1^, p53 and NOXA which leads to cell cycle arrest and AsPC-1 apoptosis	AsPC-1	(1.9 ± 0.02)	Ursolic acid (15.2 ± 0.1)		------	[71]
**29**	Inhibits proliferation of Gastric epithelial adenocarcinoma(AGS)	MRC-5 AGS SK-MES-1 J82 HL-60	(>100) (8.9 ± 0.4) (50.4 ± 3.5) (35.4 ± 2.8) (35.8 ± 4.1)	-----		Etoposide (0.33 ± 0.02) (0.58 ± 0.02) (1.83 ± 0.09) (3.49 ± 0.16) (2.23 ± 0.09)	[72]
**33**	Affinity to Mdm2 binding sites	MCF-7 U-87 MG A549 HepG2	(1.55 ± 0.08) (>100) (>100) (>100)	Ursolic acid (25.05 ± 3.17) (43.82 ± 3.88) (41.02 ± 3.77) (37.28 ± 5.02)		Doxorubicin (4.51 ± 1.12) (2.05 ± 0.22) (6.17 ± 1.17) (10.02 ± 1.67)	[75]
**36a**		A-549 MCF-7 HCT-116 THP-1 FR-2	(0.5 ± 0.05) (5.5 ± 0.08) (˂0.1 ± 0.09) (0.9 ± 0.02) (10 ± 0.04)	Ursolic acid (33 ± 0.03) (37 ± 0.07) (42 ± 0.08) (9.1 ± 0.07) (31 ± 0.08)		------	[76]
**36b**			(2.9 ± 0.05) (˂0.1 ± 0.05) (15 ± 0.06) (˂0.1 ± 0.03) (69 ± 0.05)				
**36c**			(˂0.1 ± 0.001) (˂0.1 ± 0.09) (0.3 ± 0.001) (˂0.1 ± 0.001) (˃50 ± 4.1)				
**36d**			(0.15 ± 0.01) (˂0.1 ± 0.001) (9.1 ± 1.0) (˂0.1 ± 0.001) (˃50 ± 3.9)				

n.r = not reported. Neg = negative in the concentration used.

**Table 2:** Mechanism and cytotoxicity of PTs-based pyrazole scaffolds on different cancer cells.

Hybrid Compounds	Mechanism /Pathways	Cancer Cell Lines	Hybrid Compounds (IC_50_ µM)	Reference Molecules (IC_50_ µM)		Bibliography
**41**	Induced apoptosis by hyperstimulation of macropinocytosis	Hela HepG2 HT1080 MCF-7 SK-N-MC	(18.63 ± 2.34) (27.87 ± 2.98) (26.7 ± 0.07) (25.25 ± 0.07) (28.63 ± 1.03)	Ursolic acid (43.30 ± 2.22) (34.12 ± 0.68) (39.43 ± 0.52) (57.64 ± 5.75) (67.64 ± 1.78)	----	[87]
**42**	Hindered the breast cancer cell progression by inducing apoptosis and cell cycle arrest at S and G_0_/G_1_ phase	HL-60	(0.91±0.05)	Ursolic acid (>40)	Doxorubicin (<0.63)	[88,89]
**43**	Induced apoptosis and cell cycle arrest in HepG2, HeLa, and Jurkat cell lines	Jurkat HeLa HepG2	(1.4 ± 0.2) (2.0 ± 0.3) (0.8 ± 0.05)	Betulinic acid (26.9 ± 2.2) (26.0 ± 2.1) (36.4 ± 1.5)	----	[90]
**44**	n.r		(2.3 ± 0.3) (3.0 ± 0.2) (1.7 ± 0.2)			
**45**	n.r		(11.1 ± 1.3) (3.0 ± 0.2) (2.0 ± 0.4)			
**46**	Antiproliferative, Apoptosis induction abilities correlated with upregulation of NOXA and downregulation of Bcl-xL	AsPC-1	(0.9 ± 0.01)	(˃100)	----	[91]

**Table 3:** Mechanism and cytotoxicity of PT-based piperazine scaffolds on different cancer cells.

Hybrid Compounds	Mechanism /Pathways	Cancer Cell Lines	Hybrid Compounds (IC_50_ µM)	Reference Molecules (IC_50_ µM)			Bibliography
**60**	n.r	MCF-7 Hela A549	(7.05 ± 1.45) (7.75 ± 1.09) (9.91 ± 2.11)	-----		Gefitini (17.83 ± 7.85)(15.40 ± 4.63)(11.02 ± 3.27)	[99]
**61**			(7.58 ± 1.25) (8.13 ± 1.69) (13.13 ± 4.37)				
**62**	n.r	LNCAP PC3 22RW1 Huh7 VA13	(1.12) (1.11) (0.37) (0.74) (0.19)	-----	Paclitaxel (0.01) (0.015) (0.016) (0.002) (0.001)		[100]
**63**			(n.r) (3.58) (n.r) (2.75) (n.r)				
**64**	Inhibits proliferation, Induce cell apoptosis by G1 cell-cycle arrest and through intrinsic and extrinsic apoptosis pathway.	MGC-803 HCT-116 T24 HepG2 A549 HL-7702	(9.82 ± 0.29) (18.97 ± 0.53) (13.64 ± 0.43) (5.40 ± 0.79) (11.06 ± 0.37) (>100)	Ursolic acid (27.08 ± 0.29) (38.78 ± 0.16) (29.29 ± 0.80) (30.21 ± 0.58) (35.79 ± 0.37) (>100)	5-FU (40.94 ± 0.95) (29.58 ± 1.31) (37.56 ± 0.49) (30.79 ± 0.82) (36.34 ± 0.57) (58.74 ± 2.31)		[101]
**65**	Induced cell apoptosis in MGC-803 cells.	MGC-803 Bcap-37	(2.50 ± 0.25) (9.24 ± 0.53)	Ursolic acid (24.32 ± 0.57) (28.69 ± 0.35)	Hydroxycamptothecin (>20) (>20)		[102]
**66**	Decreased the apoptosis regulator (BCL2/BAX) ratio, disrupted mitochondrial potential and induced apoptosis, and suppressed the growth of Hela xenografts in nude mice	Hela MKN45	(2.6 ± 1.1) (2.1 ± 0.3)	Ursolic acid (15.1 ± 2.7) (16.7 ± 1.4)	Cisplatin (15.1± 0.9) (2.8±0.1)		[103]
**68**	Cytotoxicity through fluorescence microscopy, annexin V assays and cell cycle analysis.	A375 A2780 HT29 MCF-7 FaDu NIH 3T3	(1.5 ± 0.4) (1.9 ± 0.3) (5.7 ± 0.5) (4.4 ± 0.7) (3.7 ± 0.6) (4.6 ± 1.0)	Ursolic acid (n.r) (11.7 ± 0.6) (10.6 ±0.7) (12.7±0.1) (n.r) (13.1±1.1)	Doxorubici (n.r) (0.01 ± 0.01) (0.9 ± 0.2) (1.1 ± 0.3) (n.r) (0.06 ± 0.03)		[104]

n.r = reported.
